# Fyn-Dependent Gene Networks in Acute Ethanol Sensitivity

**DOI:** 10.1371/journal.pone.0082435

**Published:** 2013-11-29

**Authors:** Sean P. Farris, Michael F. Miles

**Affiliations:** 1 Department of Pharmacology and Toxicology, Virginia Commonwealth University, Richmond, Virginia, United States of America; 2 Department of Neurology, Virginia Commonwealth University, Richmond, Virginia, United States of America; Mayo Clinic College of Medicine, United States of America

## Abstract

Studies in humans and animal models document that acute behavioral responses to ethanol are predisposing factor for the risk of long-term drinking behavior. Prior microarray data from our laboratory document strain- and brain region-specific variation in gene expression profile responses to acute ethanol that may be underlying regulators of ethanol behavioral phenotypes. The non-receptor tyrosine kinase Fyn has previously been mechanistically implicated in the sedative-hypnotic response to acute ethanol. To further understand how Fyn may modulate ethanol behaviors, we used whole-genome expression profiling. We characterized basal and acute ethanol-evoked (3 g/kg) gene expression patterns in nucleus accumbens (NAC), prefrontal cortex (PFC), and ventral midbrain (VMB) of control and *Fyn* knockout mice. Bioinformatics analysis identified a set of *Fyn*-related gene networks differently regulated by acute ethanol across the three brain regions. In particular, our analysis suggested a coordinate basal decrease in myelin-associated gene expression within NAC and PFC as an underlying factor in sensitivity of *Fyn* null animals to ethanol sedation. An *in silico* analysis across the BXD recombinant inbred (RI) strains of mice identified a significant correlation between *Fyn* expression and a previously published ethanol loss-of-righting-reflex (LORR) phenotype. By combining PFC gene expression correlates to *Fyn* and LORR across multiple genomic datasets, we identified robust *Fyn*-centric gene networks related to LORR. Our results thus suggest that multiple system-wide changes exist within specific brain regions of *Fyn* knockout mice, and that distinct *Fyn*-dependent expression networks within PFC may be important determinates of the LORR due to acute ethanol. These results add to the interpretation of acute ethanol behavioral sensitivity in Fyn kinase null animals, and identify *Fyn*-centric gene networks influencing variance in ethanol LORR. Such networks may also inform future design of pharmacotherapies for the treatment and prevention of alcohol use disorders.

## Introduction

Acute sensitivity to ethanol is a predictive indicator of the long-term risk of abusive ethanol drinking behavior in humans and animal models [1,2]. Fyn kinase is a non-receptor protein tyrosine kinase widely expressed in the central nervous system. Gene targeting studies show that Fyn modulates the acute sedative-hypnotic properties of ethanol [3,4] and in some studies has been shown to reduce two-bottle choice consumption in rodents [5]. Fyn modulation of NMDA or GABA receptor function [6], particularly in regard to the NR2B subunit of NMDA receptors, has been implicated as at least partially underlying Fyn modulation of ethanol behaviors. Genetic variation in Fyn is associated with alcohol dependence and alcohol related phenotypes in humans [7], supporting the premise that Fyn modulation of acute ethanol behaviors contributes to the risk for alcohol dependence. 

However, Fyn has also been shown to be important in complex aspects of neurodevelopment [8,9], myelination [10], and learning and memory [11]. Although gene-targeting studies, such as those mentioned above for *Fyn*, have been widely used to study the neurobiology of ethanol and drug abuse [12,13], the interpretation of such results is often difficult given the possible widespread molecular actions of kinases such as Fyn and the multivariate nature of complex diseases such as alcohol use disorders (AUD). Even ignoring complications such as developmental compensation in gene-targeted animals, the deletion of a single gene such as a kinase of widespread action like Fyn could modulate ethanol behaviors by triggering network-wide alterations in the function or expression of genes downstream of Fyn, in addition to mechanisms related to direct targets of *Fyn* phosphorylation. 

The mesolimbocortical dopaminergic reward pathway, comprised of the prefrontal cortex (PFC), nucleus accumbenes (NAC), and ventral midbrain (VMB), is activated by acute ethanol and other drugs of abuse [14]. Baseline differences or drug-induced alterations in gene expression within the mesolimbocortical dopamine pathway may play an important role in the transition from initial drug exposure to the development of dependence [15,16]. Previous research from our laboratory has shown divergent basal and acute ethanol-evoked patterns of gene expression across the dopamine reward pathway that may contribute to acute ethanol behavioral sensitivity [17,18], and we have shown that altered expression of an ethanol-responsive gene (*Clic4*) in medial prefrontal cortex can modify ethanol loss-of-righting reflex (LORR) [19]. Therefore, it is our hypothesis that altered expression or function of Fyn kinase may produce network level changes in gene expression within the mesolimbocortical dopamine pathway, thus providing an important mechanism modifying behavioral responses to acute ethanol. 

Using expression profiling we sought to define Fyn-dependent gene networks underlying ethanol behavioral traits; with emphasis on ethanol-induced LORR due to the reproducible association of Fyn kinase genotype with this behavioral phenotype [3-5,20]. Expression profiling has been previously employed to determine downstream signaling mechanisms altered by single gene knockout animals exposed to acute [21] and chronic ethanol exposure [22]. However, a study of ethanol-responsive gene expression patterns in mice carrying a null mutation for *Fyn* has not been reported. Characterizing such gene expression patterns is also important for understanding the neurobiology of Fyn, given the fundamental role of this kinase in development, receptor function, behavior, and regulation of numerous signaling cascades. 

Our expression profiling and bioinformatics results suggest multiple Fyn-related mechanisms, especially those affecting a network of myelin-related gene expression within the medial PFC, as contributing to the sedative-hypnotic properties of acute ethanol. Variation in the expression of these Fyn-dependent gene networks may be critical molecular endophenotypes affecting the behavioral level of response to acute ethanol, and subsequently, the long-term risk for alcohol use disorders.

## Materials and Methods

### Ethics Statement

All procedures were approved by Virginia Commonwealth University Institutional Animal Care and Use Committee under protocol number AM10332 and followed the NIH Guide for the Care and Use of Laboratory Animals (NIH Publications No. 80–23, 1996).

### Animal microdissection and acute ethanol administration

Animals were treated according to protocols for animal care established by Virginia Commonwealth University and the National Institute for Health. Adult male B6129SF2/J and B6;129S7-Fyn^tm1Sor^/J mice obtained from Jackson Laboratories at 12 weeks of age and were housed 4-5 per cage with *ad libitum* access to water and standard rodent chow (#7912, Harlan Teklad, Madison, WI) on a 12 hr light/dark cycle with Harlan Sani-chips bedding (#7090A). Mice were habituated to the animal facility for 1 week prior to initiating experiments.

Control and Fyn-null mice (n=18 of each genotype) were administered intraperitoneal injections of saline for 3 days to habituate them to the injection process; on day 4 mice received either an injection of saline (n=9 of each genotype) or 3 g/kg (20% v/v) of ethanol (n=9 of each genotype), a sedative-hypnotic dose [23]. Animals were sacrificed by cervical dislocation and decapitation at a 4-hour time-point. Our laboratory has previously found that a 4-hour time point captures a spectrum of early, intermediate, and late gene expression responses to ethanol (Ravindranathan and Miles, unpublished). Microdissection of individual brain regions was conducted exactly as described previously [17]. Brain regions were individually frozen immediately with liquid nitrogen, and subsequently stored at -80°C until isolation of total RNA. 

Tissue pooled from three mice of the same genotype/treatment group was homogenized in PureZol Reagent (Bio-Rad Laboratories, Hercules, CA) using a Tekmar homogenizer, and total RNA was isolated with the Aurum Total RNA fatty and Fibrous Tissue Kit according to the manufacturer’s instructions. RNA concentration was determined by absorbance at 260 nm, and RNA quality was analyzed by 260:280 nm absorbance ratios and electrophoretic analysis (Experion; Bio-Rad Laboratories, Hercules, CA). Double-stranded cDNA and biotin-labeled cRNA was synthesized using reagents and protocols from the microarray manufacturer (Affymetrix, Santa Clara, CA). 

### Microarray Hybridization and Scanning

Biological replicates (n=3) from pooled samples within each treatment group and genotype were hybridized to individual microarrays for prefrontal cortex (PFC), nucleus accumbens (NAc), and ventral midbrain (VMB) areas (n=36 total microarrays). Arrays for a single brain region were processed together in one day, using a supervised randomization of samples in order to minimize potential batch effects. Labeled cRNA samples were analyzed on oligonucleotide arrays (Affymetrix Mouse Genome 430 2.0 arrays) that contain ~36,000 genes and expressed sequence tags. Hybridization, washing, staining and scanning were performed according to manufacturer protocols (Affymetrix).

### Microarray Data Analysis

Microarray data were initially processed using GeneChip Operating Software v4.1 (GCOS, Affymetrix). Arrays were normalized to a median total hybridization intensity (target average intensity, 190) and quality was assessed by array scaling factor (<3), 3’-5’ expression ratios for control probes, absent/present calls (%present ≥ 55% for all arrays) and by inspection of pairwise Pearson correlations and scattergrams. Arrays passing quality control were processed for any potential hybridization batch effects [24]. Processed arrays were initially filtered for Absent/Present calls [25] to eliminate probesets consistently called “absent” across all samples, and then subjected to S-score analysis [26]. The S-score algorithm, developed in our laboratory for analyzing Affymetrix arrays was applied to compare hybridization signals between two arrays from differing treatment samples. An S-score of |2| corresponds to a p = 0.0455, uncorrected for biological variability or multiple comparisons. S-scores were generated using all of the pairwise comparisons between ethanol treated controls and *Fyn* knockout mice, as well as basal differences between genotypes. The average S-scores from all individual subject pairwise comparisons were used to represent a single biological replicate in downstream analyses. Within each brain region and treatment, S-scores were divided by the greater of 1 or the group standard deviation from same/same comparisons to reduce the contribution of biological or technical noise, as described previously [17]. Array data has been deposited in the Gene Expression Omnibus database (www.ncbi.nlm.nih.gov/geo/) with accession number GSE 9028.

A one-class statistical analysis of microarrays (SAM) was used to within each treatment group/brain region to determine those genes with S-scores significantly different from 0 [27]. Differences in ethanol-regulation of gene expression were determined using 2-class SAM within each brain region (i.e. Fyn KO vs CTL). Within each SAM analysis S-scores were filtered for an average ≥ 1.5 or ≤ -1.5 (composite significance, p < 0.01) to focus on the most biologically robust results. All SAM analyses were filtered for a median false-discovery rate (FDR) ≤ 5%. Significant genes/probesets were subsequently subjected to k-means clustering [28] to determine coordinately-regulated gene expression patterns, and demonstrate brain-region, treatment, and genotype specific differences in gene expression.

### Bioinformatics Analysis Of Microarray Data

Toppgene Suite [29] and ErmineJ [30] were used for data exploration of functional classification among gene expression profiles using gene ontology categories, mouse phenotype data, and public pathway databases. ToppFun (functional enrichment analysis within Toppgene Suite) calculations were set to a 5% false discovery rate, with gene limits of *n* ≥ 3 and ≤ 300 to identify representative *a priori* ontological categories. ErmineJ gene set analysis was implemented for over-representation analysis (ORA) using the best scoring gene replicate and a gene score threshold of 1.5; reported p-values were corrected for the ErmineJ default of a 10% FDR. For ErmineJ analysis a custom extended myelin-associated gene set was constructed as an additional GO category based upon repeated microarray data cluster associations for myelin genes from our laboratory (Farris and Miles, unpublished data and see 17). Additional pathway analysis of statistically significant genes was done using Ingenuity Pathway Analysis (www.ingenuity.com), a curated bioinformatics resource for discovery of gene-gene interaction networks based on literature association, biological function, and cell-signaling mechanisms. 

Fyn-centric correlation networks were constructed using the intersection of Fyn knockout data herein and *Fyn* expression correlations (Pearson correlation p-value ≤ 0.01) across microrarray datasets previously generated by our laboratory from PFC of the BXD [18] and LXS (Miles and Johnson, unpublished data) recombinant inbred mouse lines (datasets GN135 and GN130, respectively, publically available at www.genenetwork.org). Due to the redundant design of Affymetrix microarrays with multiple probesets representative of a single gene, all probesets representing a single gene were considered for analysis with multiple probesets represented as a single node with the removal of duplicate edges (i.e. correlations) between two genes and self-loops among the same gene. Visualization of gene correlation networks were rendered in Cytoscape (www.cytoscape.org) [31]. Resulting Fyn-centric correlation networks were submitted to GeneMANIA (genemania.org) [32] to independently assess protein and genetic interactions, pathways, co-expression, co-localization and protein domain similarity as well as identify potential candidates not directly identified from microarray analysis. 

## Results

### Gene Expression Pattern Changes In *Fyn* Null Mice

**Figure 1:**
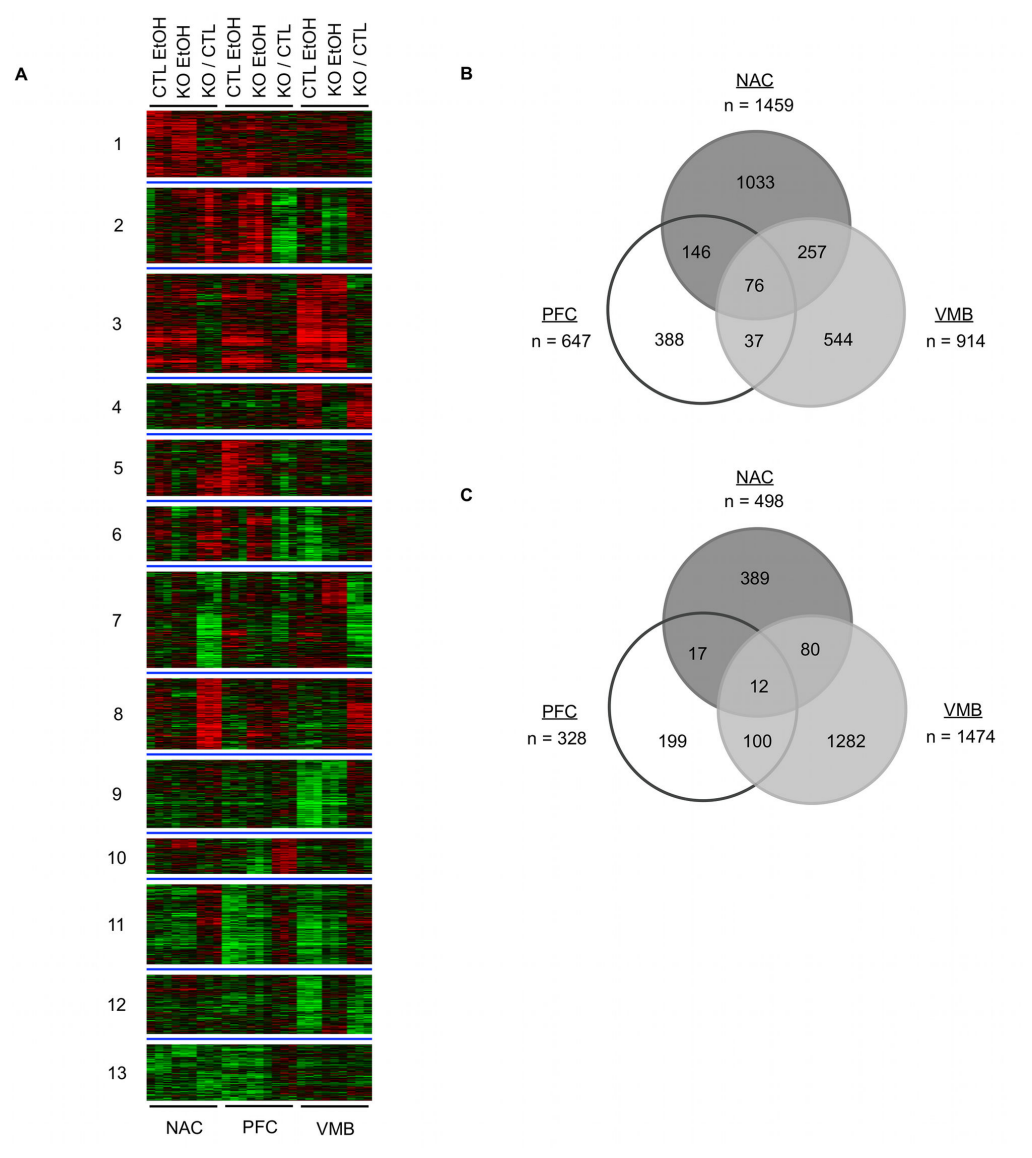
Basal and ethanol-responsive gene expression differences between *Fyn* knockout mice and controls. (A) k-Means cluster analysis of differential gene expression for S-scores; green = decreased relative expression, red = increased relative expression, black = no change in expression (Control EtOH = CTL EtOH, KO EtOH = Fyn Knockout EtOH, KO / CTL = Fyn Knockout Saline / Control Saline). (B) Venn diagram of overlapping and non-overlapping genes differentially expressed between saline treated *Fyn* knockout mice and controls. (C) Venn diagram of overlapping and non-overlapping genes differentially expressed between acute ethanol (3 g/kg) treated *Fyn* knockout mice and controls. (NAC - Nucleus Accumbens, PFC - prefrontal cortex, and VMB - ventral midbrain).

Genome-wide expression profiling across the mesolimbocortical dopamine pathway of saline or ethanol-treated male *Fyn* null mice was conducted in order to characterize the molecular mechanisms underlying altered behavioral responses to ethanol in this mutant mouse line. We performed whole-genome expression profiling in the presence and absence of an acute anesthetic dose of ethanol (3 g/kg) since ethanol LORR is the most reproducible ethanol phenotype altered in Fyn null animals. Direct or indirect changes in gene expression resulting from elimination of *Fyn* were determined through one-class and two-class SAM analysis of S-scores for either basal or ethanol-responsive gene expression. Multivariate analysis using k-means clustering identified region specific patterns of gene expression (Figure 1A; Table S1); basal and ethanol-treated gene expression across each of the three brain regions was combined to increase the statistical power of k-means clustering. As expected from the overall expression distributions (Figure 1B & 1C), most clusters showed basal (KO/CTL) differences that were unique to a single or two brain regions. Several clusters showed possible trends toward differences in ethanol responses, as well as basal expression changes with the Fyn KO, particularly in the VMB (see clusters 2, 3, 4, 6, 9, 11 and 12 for VMB). Generally, differences in basal and ethanol-responsive gene expression were distributed unevenly among the three brain regions with basal alterations ranging NAC > VMB > PFC (Figure 1B) and ethanol- responses varying in the order of VMB > NAC > PFC (Figure 1C). 

### Bioinformatic Analysis Of Basal Expression Changes In *Fyn* Null Mice

In order to assess the potential biological significance of basal expression differences due to a null mutation for *Fyn* we performed a functional over-representation analysis for each individual brain-region using the ToppGene Suite web-portal (FDR ≤ 0.05, Table 1 Basal Gene Ontology Categories; full-set available as Table S2). Evaluation of functional changes may be important for determining the role of Fyn within different brain-regions and subsequent phenotypic impact of a *Fyn* null mutation. Although some categories are certainly redundant, the overall number of differentially expressed genes for each brain-region was disproportionate to the number of significant functional groups. PFC had the smallest change in total number of genes among the three brain structures; however, the PFC also showed the largest number of significant functional categories. 

**Table 1:** Gene Ontology Over-Representation Analysis of Basal Gene Expression.

**Brain Region**	**ID**	**Gene Ontology Name**	**P-value**	**Query #**	**Genome #**
**NAC**	8022	protein C-terminus binding	0.000019	34	186
	19911	structural constituent of myelin sheath	0.000019	5	5
	5158	insulin receptor binding	0.000019	11	33
	22843	voltage-gated cation channel activity	0.046044	27	150
	7612	learning	0.007789	21	78
	50890	cognition	0.025714	30	155
	1508	regulation of action potential	0.025714	23	103
	7611	learning or memory	0.026309	28	144
	19228	regulation of action potential in neuron	0.026309	20	85
	1505	regulation of neurotransmitter levels	0.026309	27	138
	71375	cellular response to peptide hormone stimulus	0.046517	39	246
**PFC**	35254	glutamate receptor binding	0.000125	9	29
	19900	kinase binding	0.000125	28	297
	19901	protein kinase binding	0.000125	25	253
	8022	protein C-terminus binding	0.000125	20	186
	19911	structural constituent of myelin sheath	0.000125	4	5
	5250	A-type (transient outward) potassium channel activity	0.000125	3	3
	22843	voltage-gated cation channel activity	0.000125	16	150
	5516	calmodulin binding	0.000125	16	150
	17124	SH3 domain binding	0.000125	14	122
	3924	GTPase activity	0.000125	20	226
	17075	syntaxin-1 binding	0.000125	5	15
	5057	receptor signaling protein activity	0.000125	13	114
	5249	voltage-gated potassium channel activity	0.000126	12	99
	15271	outward rectifier potassium channel activity	0.000142	4	9
	15276	ligand-gated ion channel activity	0.000152	14	132
	22834	ligand-gated channel activity	0.000152	14	132
	15631	tubulin binding	0.049736	14	132
	48167	regulation of synaptic plasticity	0.000742	16	86
	50804	regulation of synaptic transmission	0.004285	22	184
	51969	regulation of transmission of nerve impulse	0.007439	22	197
	31644	regulation of neurological system process	0.007439	23	215
	6813	potassium ion transport	0.007439	21	185
	48169	regulation of long-term neuronal synaptic plasticity	0.028724	8	31
	7215	glutamate signaling pathway	0.038472	8	33
	18107	peptidyl-threonine phosphorylation	0.038472	8	34
	32886	regulation of microtubule-based process	0.038472	11	68
	31111	negative regulation of microtubule polymerization or depolymerization	0.038472	7	26
	1505	regulation of neurotransmitter levels	0.038472	16	138
	7026	negative regulation of microtubule depolymerization	0.038472	6	18
	31114	regulation of microtubule depolymerization	0.038472	6	18
	14047	glutamate secretion	0.039458	8	36
	70507	regulation of microtubule cytoskeleton organization	0.039458	10	58
	18210	peptidyl-threonine modification	0.043982	8	37
**VMB**	31644	regulation of neurological system process	0.006772	29	215
	71845	cellular component disassembly at cellular level	0.016133	30	245
	22411	cellular component disassembly	0.016133	30	249
	51969	regulation of transmission of nerve impulse	0.034958	25	197

Functional over-representation analysis of basal (saline-treated) gene expression differences between control and *Fyn* null mice within NAC, PFC, and VMB. Shown are gene ontology categories for ‘Molecular Function’ and ‘Biological Process’ with P-values corrected for a 5% FDR (A list of all categories is included in Table S2).

#### Prefrontal Cortex

The medial PFC has important modulatory effects on the dopaminergic reward system through glutamatergic feedback to the nucleus accumbens and ventral midbrain [33]. Functional over-representation analysis implicated *glutamate receptor binding* (GO:0035254; P-value = 0.000125) and *glutamate signaling pathway* (GO:0007215; P-value = 0.038472), with a decrease in the NMDA receptor obligatory subunit *Grin1* and increase in *Grin2b* expression. Other genes in these functional groups that showed basal expression differences in the *Fyn* KO, included *Dlg4* (Psd-95) and *Homer1*, which both had decreased expression in null mice. Basal variation in this system within PFC is important due to prior evidence of ethanol-mediated long-term facilitation of glutamate receptors containing the NR2B (*Grin2b*) subunit, which is phosphorylated by Fyn kinase in a brain region specific manner [34]. Glutamatergic receptors can also be regulated by other signaling proteins, including H-ras and Src [35]. Of note, *Fyn* null mice exhibited increases in basal transcript abundance of *Hras* and *Src* (Table S1), possibly a compensatory response to loss of Fyn activity. *Hras* and *Src* were contained in several over-represented ontological categories including *regulation of synaptic plasticity* (GO:0048167, P-value = 0.000742) and *regulation of synaptic transmission* (GO:0050804, P-value = 0.004285). NMDA subunit receptor composition is altered following exposure to ethanol with a relative increase in the NR2B subunit within the membrane. This functional change in receptor subunit composition is due to H-Ras activation and inhibition of Src kinase [35]. 

#### Nucleus Accumbens

Clusters 7 and 8 (Figure 1A) showed strong inverse relationships in basal gene expression within NAC. Although the NAC is important in the neurobiology of addiction, it also has a recognized role in learning and memory [36]. Gene ontology analysis of NAC showed a broad range of categories centered on the abnormal expression of genes in Fyn knockout mice involving neuronal transmission and biological processes related to learned behavior (Table 1; Table S2). In support of these findings, *Fyn* knockout mice have been previously reported to exhibit abnormal spatial learning [37] and hyper-responsiveness to fear inducing stimuli [38]. Our expression results suggest that Fyn-dependent gene expression in NAC may contribute to these learning/memory behavioral differences in *Fyn* null mice.

#### Ventral Midbrain

Tissue from the Ventral Midbrain (VMB), encompassing the ventral tegmental area, had several clusters differentially expressed between knockouts and controls (see Clusters 4, 7, 8, and 12). The overall functional impact of these expression patterns as a whole for VMB was less obvious compared to PFC and NAC, with significant ontologies related to altered nervous system function (Table 1. GO: 0031644 & GO:0051969) and the breakdown of cellular components (Table 1. GO: 0071845, GO:0022411). 

### Myelin-Associated Gene Expression

**Figure 2:**
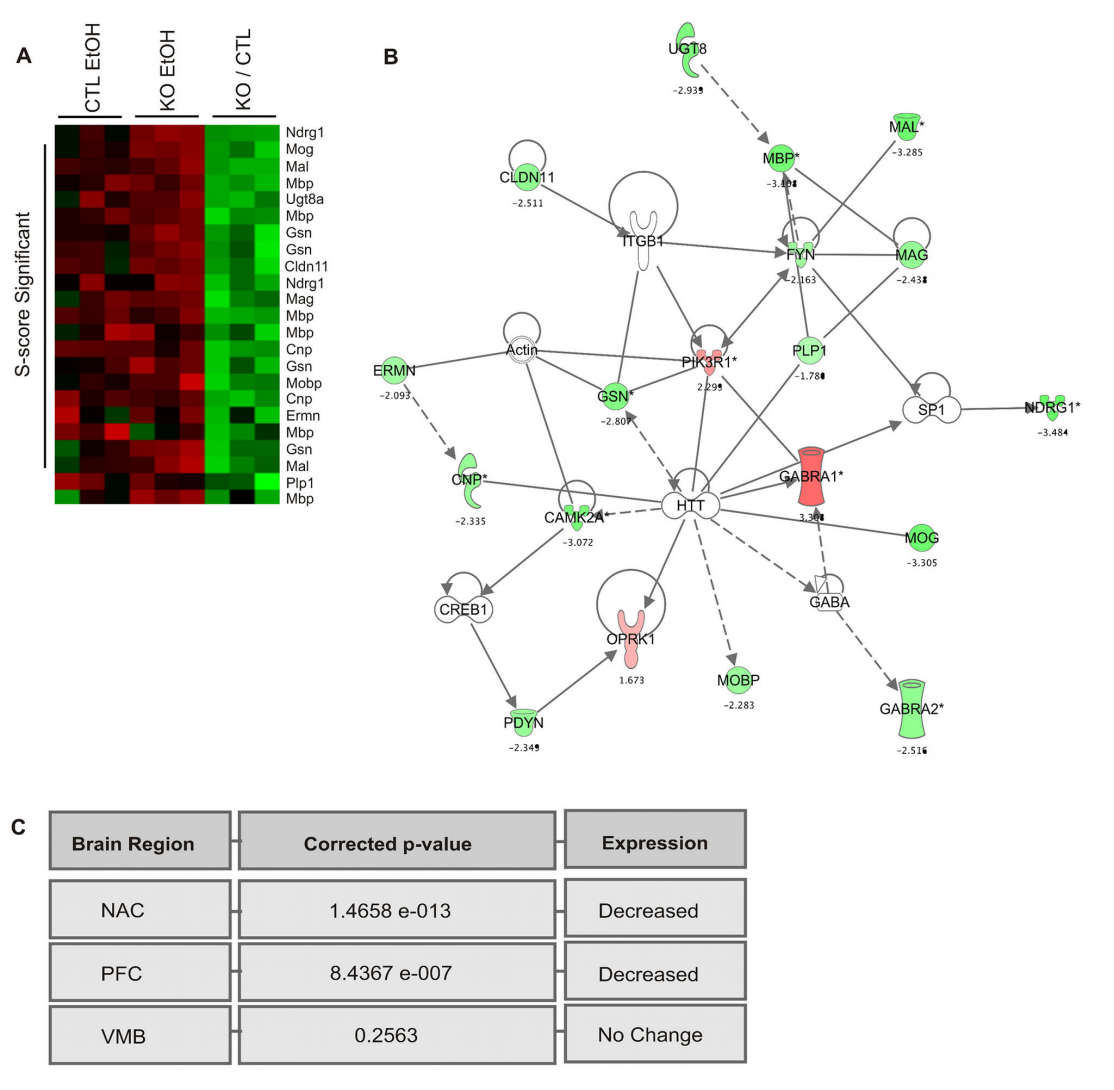
Over Representation Analysis (ORA) of Myelin-Associated Gene Expression. (A) ErmineJ heatmap of NAC gene expression showing a coherent decrease for myelin-related basal gene expression in *Fyn* knockouts compared to control. (B) Ingenuity Pathway Analysis (IPA) of myelin-related genes and literature associations; numbers shown are mean S-score of three biological replicates. Red indicates increased expression in *Fyn* knockout vs controls and green fill indicates decreased expression in the *Fyn* knockout. (C) Table display of ErmineJ corrected p-values (10% FDR) for myelin gene expression in NAC, PFC, and VMB.

As suggested by previously published research with a less extensive expression analysis [39,40], our results detected a significant decrease of myelin-associated gene expression in *Fyn* knockout mice. The 76 genes with altered basal expression overlapping across all three brain regions (Figure 1B) were over-represented for structural constituent of the myelin sheath (GO: 0019911; P-value = 0.000105 uncorrected for multiple comparisons). This ontological category, as defined by the Gene Ontology Consortium (http://www.geneontology.org/) is limited by the inclusion of only 5 myelin genes (*Mal*, *Mbp*, *Plp1*, *Tspan2*, *Mobp*), two of which (*Mbp* and *Plp1*) were decreased in *Fyn* null animals on our microarray analysis. Therefore, we extended this study to include other myelin-associated genes by using ErmineJ [30] for over-representation analysis. We tested the over-representation of a dozen myelin-associated genes (see Methods) based on their absolute expression within each individual brain region (Figure 2A, FDR ≤ 10%). As also shown in Figure 2C (and see Table 1 and Table S2), basal expression for these myelin genes were decreased in *Fyn* null animals within the NAC and PFC, but not the VMB, were significantly over-represented for myelin-related genes. Ingenuity Pathway Analysis of the NAC dataset (Figure 2B) further showed the functional relationship between Fyn kinase and myelin, as well as other potentially related genes such as myelin basic protein expression factor 2 repressor (*Myef2*). Our prior genomic studies also identified differential basal expression of myelin genes between C57BL/6J and DBA/2J mice [17], which show divergent behavioral responses to acute ethanol. Together these results suggest that a hypomyelination phenotype for *Fyn* knockout animals may be important for the interpretation of ethanol behavioral phenotypes in this model system. More generally, these data may suggest that coordinate differential expression of a myelin-related gene cluster and associated signaling mechanisms could contribute to acute ethanol behavioral responses. 

### Ethanol-Responsive Gene Expression

In addition to basal differences in gene expression, differences in ethanol-evoked signaling events may contribute to altered ethanol behavioral phenotypes in Fyn kinase knockout animals. Using gene expression as a surrogate measure of signaling mechanisms altered by acute ethanol we performed microarray analysis of NAC, PFC, and VMB from knockout and control animals 4 hours post an acute ethanol exposure (Figure 1A). A 4-hour time-point was chosen due to prior studies from our laboratory showing that this time-point captured a spectrum of early, intermediate, and late gene expression responses to ethanol ([17] and data not shown). We chose a 3 g/kg (i.p.) dose of acute ethanol that has been previously used in regards to behavioral genetics of ethanol’s sedative-hypnotic effects [23]. 

**Figure 3:**
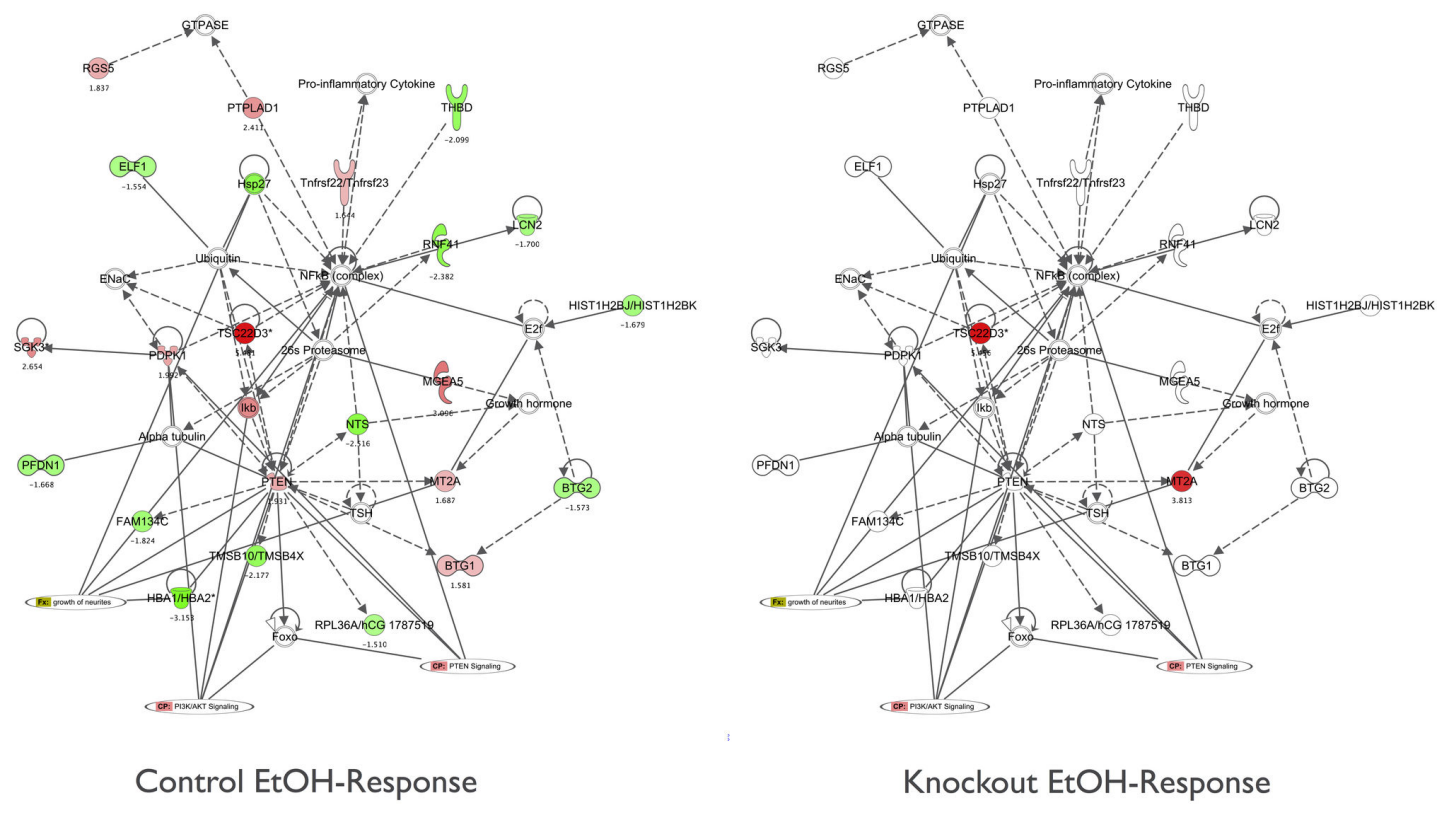
*Fyn* Knockout Animals Have Altered Ethanol-Responsive Gene Networks. Ingenuity Pathway Analysis from two-class SAM filtering for network-level differences in ethanol-responsive gene expression for the Nucleus Accumbens in control (left panel) versus Fyn knockout (right panel) mice. Genes labeled in green are down-regulated by acute ethanol; Red labeled genes are up-regulated by acute ethanol. Numbers shown are the mean S-score for 3 biological replicates. Data for additional brain regions are shown in Figure S1.

Global changes in gene expression occurring similarly across all individual brain regions due to acute ethanol exposure were captured using a one-class SAM analysis and included in k-means clustering for visualization (Fig 1A, e.g. cluster 3). Differences in ethanol-responsive expression were determined using a two-class SAM analysis within each brain structure (Figure 1A & 1C). Unlike our previous studies between B6 and D2 mice, although there were a significant number of differences in ethanol responsive genes between controls and *Fyn* null animals in each brain region (Figure 1C), region-specific ethanol patterns were difficult to discern visually using k-means clustering. This was likely due to large number of changes in expression from the higher dose of acute ethanol (i.e. one-class SAM), resulting in more quantitative rather than qualitative differences in ethanol responses between the control and null animals, as well as the prominent differences in overall basal abundance due to loss of *Fyn* (Figure 1B). Subsequently we further analyzed these resulting acute ethanol expression differences for separate brain areas using a multivariate bioinformatics approach described in Materials and Methods. Unlike over-representation analysis of basal differences (Table 1), gene ontology analysis of ethanol-responsive expression at a 5% FDR yielded few significant groups related to gene function (not shown; see Table S3 for total gene ontology results). This suggested that basal variation of system-wide changes in expression (i.e. glutamate receptor function and myelin-associated gene expression) is a stronger predictor of acute ethanol sensitivity in the Fyn kinase knockout mouse. Alternatively, it could be that our use of a 5 % FDR is overly conservative in determining ontological groups related to gene function following a transient event such as with a single ethanol exposure on this specific genetic background. However, literature association analysis using curated resources from Ingenuity Pathway Analysis (IPA) and GeneGo did suggest network-level differences in ethanol-regulated gene expression between knockout and control animals (Figure 3 and Figures S1A and S1B) as discussed below. 

#### Nucleus Accumbens

The ethanol-responsive IPA network in Figure 3 depicts a set of functionally related genes regulated by acute ethanol in control animals essentially blocked in their mRNA regulation by ethanol in animals carrying a *Fyn* null mutation. Members of this network are involved in the phosphoinositide 3-kinase (PI3K), AKT and phosphatase and tensin homolog (PTEN) signaling pathways. Acute ethanol administration causes a coordinate activation of the AKT signaling pathway within NAC that when inhibited leads to a reduction in binge drinking and ethanol self-administration [41]. Taken together, these published data and our array results suggest that this Akt/PI3k/Pten network may function both in modulating basal ethanol seeking behavior and Fyn-dependent adaptive responses to ethanol exposure. 

#### Prefrontal Cortex

Several genes related to axonal guidance, long-term potentiation and synaptic transmission were regulated by acute ethanol within PFC of *Fyn* null animals, but non-responsive in controls in the network displayed in Figure S1A. For example, expression of brain derived neurotrophic factor (Bdnf) and synaptophysin (*Syp*) were decreased while *Kcnma1*, *Arl6ip5* and *Camkk2* were increased by acute ethanol in PFC of *Fyn* null animals (Figure S1A, lower panel) but were not regulated by ethanol in controls (Figure S1A, upper panel). This suggests that, in contrast to the Akt/PI3k/Pten cascade in NAC mentioned above, signaling mechanisms regulating these members of the Figure S1A gene network were more sensitive to ethanol in the Fyn null animals than in controls.

Altered ethanol regulation of *Bdnf* in the *Fyn* null animals is a particularly interesting finding given the role of BDNF in synaptic plasticity and documentation of *Bdnf* regulation by ethanol and other drugs of abuse. For example, increased abundance of BDNF in PFC following cocaine withdrawal facilitates activity induced long-term potentiation [42]. Acute ethanol exposure can lead to increased levels of *Bdnf* mRNA in striatum [17], and altered voluntary ethanol intake occurs in mice with dysregulation of BDNF expression in the cortico-striatal network [43]. 

#### Ventral Midbrain

Top-ranked IPA networks for ethanol-responsive differences within VMB were broadly related to RNA processing, cell signaling, and neurogenesis (Table S2). For example, ethanol regulation of *Epha7* gene expression was in opposite directions between Fyn null mice and controls (Figure S1B) and multiple other members of this same network showed diminished responses to ethanol with Fyn deletion. Ephrin receptors are one of the largest families of tyrosine kinases with an important role in neurodevelopment and synaptic function [44]. Epha7 phosphorylates Erk [45], which regulate the expression of genes involved in numerous processes including neuronal development and plasticity [46]. As shown in Figure S1B, multiple genes interacting with Erk signaling were regulated by acute ethanol with VMB of control animals (upper panel), but not with *Fyn* null mice (lower panel). 

However, the most striking feature of the VMB network shown in Figure S1B was that acute ethanol decreased expression for a large category of genes related to RNA processing in controls, but the same genes were unresponsive in *Fyn* knockout animals (Figure S1B, lower panel). Similarly, the mRNA expression of *Dicer1* was increased in control animals, but not regulated in knockouts. Dicer is critical in mouse development [47], processing of microRNAs that in turn regulate mRNA expression [48], and has a functional role in midbrain dopaminergic neurons and associated behaviors [49]. Our analysis suggests that signaling events regulating a VMB RNA processing gene network are modulated by ethanol in a Fyn kinase-dependent manner. Whether this molecular pathway may regulate acute ethanol sensitivity or adaptive responses to chronic ethanol remains to be determined. However, RNA processing was also identified in a systems genetic analysis of alcohol sensitivity in *Drosophila melanogaster* [50]. 

The IPA networks discussed above suggest significant network-level brain region selective effects of Fyn deletion on ethanol-responsive gene expression, in contrast to the more subtle brain region differences of ethanol responses noted in the cluster analysis of Figure 1. This is further emphasized in Figure S1C where summation of S-scores are shown to be strikingly different between controls and *Fyn* null animals across the PFC, NAC and VMB networks.

### Fyn-Related Gene Network Analysis For LORR

**Figure 4:**
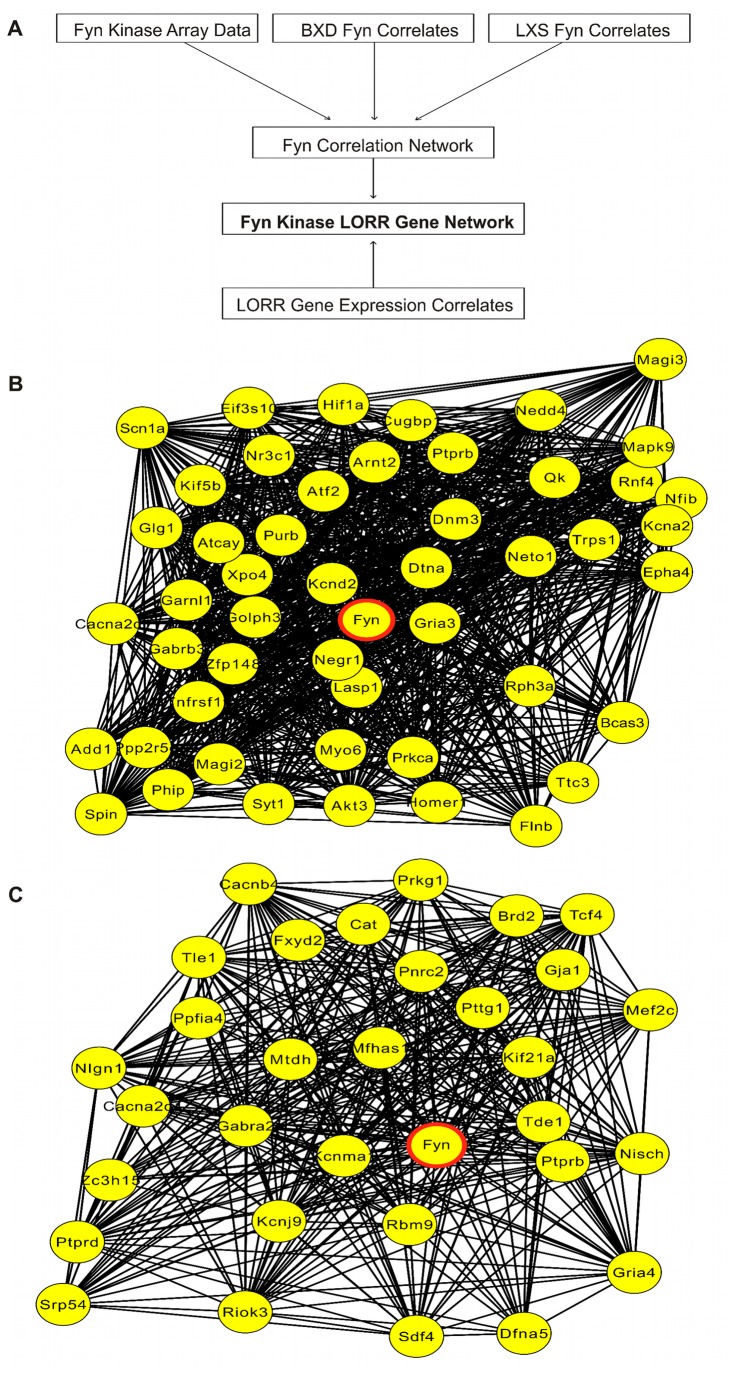
Fyn-LORR Correlation Network within PFC. Network analysis of concurrent variation of gene expression amongst PFC *Fyn* knockout array data, *Fyn* expression correlates across the BXD RI PFC, *Fyn* expression correlates across the LXS RI PFC, and LORR – BXD gene expression correlates in PFC. (A) Diagram of general approach used to define a Fyn-dependent gene network for the loss of righting reflex behavior. (B) Basal Fyn-dependent gene network showing correlations among BXD RI RMA saline dataset (node connections indicate correlation p-value ≤ 0.01). (C) Ethanol-responsive Fyn-dependent gene network showing correlations among BXD RI S-score dataset (p-value ≤ 0.01). Red circle indicates position of *Fyn*.

Null mutations of *Fyn* increase duration of the loss of righting reflex (LORR) response to acute ethanol [3-5]. In order to further assess molecular mechanisms for *Fyn* involvement in ethanol LORR, we compared our microarray analysis of *Fyn* null mice with gene expression correlates of LORR or *Fyn* expression across panels of recombinant inbred mice (Figure 4A). PFC microarray data was used for this analysis due to its availability and since the PFC dissections include adjacent motor cortex [17] and we have shown that selective modulation of PFC gene expression can alter ethanol LORR [19]. As described in Methods, *Fyn* gene expression correlates (Pearson correlation p-value < 0.01) were derived in PFC across the BXD (n = 29) [18] and LXS (n = 42) recombinant inbred mouse lines. Additionally, we identified BXD PFC gene expression correlating with a previously published LORR study across BXD RI mice (GeneNetwork ID: 10589 [51]). Overlapping data from these three microarray correlation gene sets together with the basal expression differences identified here in the Fyn null mice (Figure 1, Table S1) allowed definition of a Fyn-LORR gene network (Figure 4A; Figure S2).

**Figure 5:**
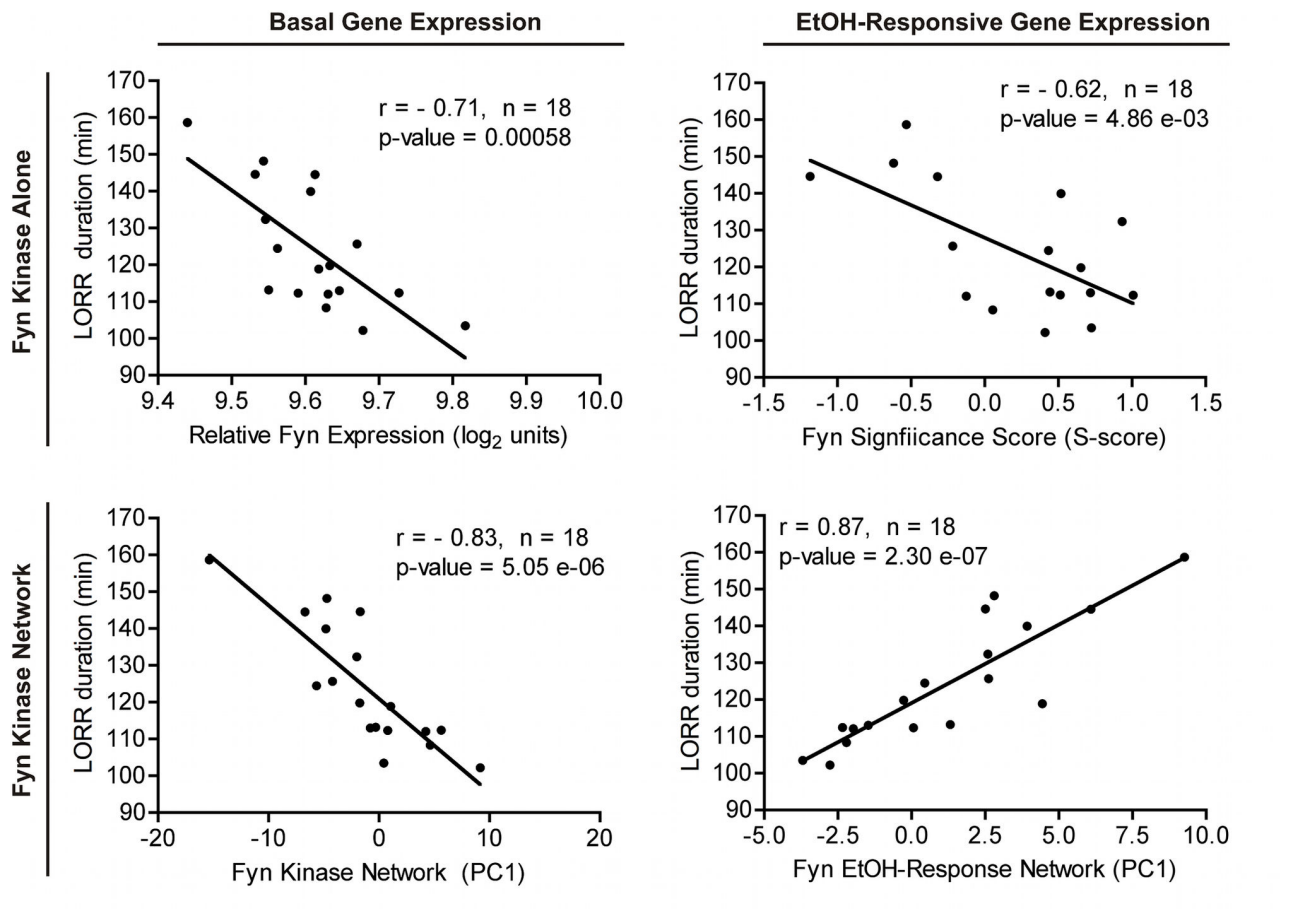
*In* *silico* correlation analysis of Fyn, Fyn gene networks and LORR behavior. Pearson product moment correlations analysis of basal saline (RMA) gene expression (right panels) and ethanol-responsive (S-score) expression (left panels) versus ethanol LORR behavior (Rodriguez et al., 1995; WebQTL Record ID: 10589). Expression for *Fyn* (upper panels) or Fyn-dependent networks (lower panels) from Figure 4B and 4C are plotted versus LORR. For Fyn-dependent networks, the first principal component (PC1) for expression of all genes in the network was used for correlations with LORR. PC1 values are relative and do not indicate overall positive or negative correlations with LORR.

Analysis of basal gene expression from the *Fyn* null mice by the scheme mentioned above identified a network of 50 highly inter-correlated genes (Figure 4B, Table S4); the first principal component of these 50 genes significantly correlated to LORR (r = -0.83, p-value = 5.05 E-06; Figure 5, left lower panel). Functional over-representation analysis of this gene network highlighted ion channel activity, function/localization to the post-synaptic density and dendritic spines, and regulation of action potentials (Table S5) as over-represented groups. *Neto1*, *Kcnd2*, *Dnm3*, *Gria3*, and *Homer1* are all localized to the post-synaptic density and also may play a role in modulating synaptic N-methyl-D-aspartic (NMDA) acid receptor function. *Neto1* knockout mice have abnormal long-term potentiation, learning, and memory due to altered NMDAR subtype abundance [52]. *Kcnd2*, also known as *Kv4.2*, is a voltage-gated potassium channel that regulates spontaneous NMDAR activation and downstream calcium signaling mechanisms [53]. *Dnm3* is a binding partner of *Homer1* [54] a scaffolding protein associated with type1 metabotropic glutamate receptors and the NMDAR complex. The ionotropic glutamate receptor *Gria3* is a member of the AMPA-receptor family, which facilitate fast excitatory synaptic transmission. Thus, although the literature suggests a dominant role of NR2B NMDA receptor subunit function in Fyn modulation of ethanol behaviors, our genomic analysis results suggest that the Fyn-dependent behavioral phenotypes may also be modulated through several genes related to glutamate function and possibly additional mechanisms as well (see discussion below on myelin). 

In addition to the glutamate-related ion channels and support proteins mentioned above, *Kcna2*, *Gabrb3*, *Cacna2d1*, *Scn1a* were present in Figure 4B, suggesting Fyn kinase signaling is interconnected with regulation of gene expression for multiple ion channels. The beta-3 subunit of GABA-A receptor has been previously reported to be functionally altered in *Fyn* kinase knockout mice, contributing to reduced LORR sensitivity with etomidate, a beta-2/beta-3 selective compound [55]. *Scn1a* is expressed in the nodes of Ranvier of motor neurons, regulating the propagation of action potentials [56], and resides on Chr 2 within a QTL for alcohol preference [57], although no actual link to ethanol behaviors has been shown for this gene.

The Fyn-dependent basal gene network was also significantly over-represented for ‘abnormal white matter morphology’ (p-value = 0.000003; MP:0008026), and as shown in Figure 2, white matter related genes were significantly decreased in the PFC of *Fyn* knockout mice. *Fyn* expression was also correlated to Quaking (*Qk*) (Figure 2B), which is known to regulate the transport of *Mbp* mRNA from the nucleus [58]. *Fyn* specifically regulates the activity of Quaking through phosphorylation of tyrosine residues for binding *Mbp* mRNA within the C-terminal domain of Quaking [59]. Thus, a Fyn-dependent, myelin-related gene expression network altered in Figure 2B could contribute to Fyn modulation of ethanol LORR. 

In addition to differences in basal gene expression that may contribute to variation in acute ethanol sensitivity of *Fyn* null mice, the LORR behavior may also be influenced by differences in acute ethanol-evoked signaling events. Again using S-score analysis as a measurement of ethanol-responsive gene expression (i.e. EtOH/Saline), we used the experimental design outlined in Figure 4A, with ethanol-responsive gene expression values from the various datasets for correlation to Fyn or LORR, to identify a set of 32 distinct genes across the four different datasets (Figure 4C, Table S6). The first principal component of the ethanol-responsive network was significantly correlated to the LORR (r = 0.87, p-value = 2.30 e-07), greater in magnitude than for Fyn kinase ethanol-response alone (Figure 5, right panels), suggesting that signaling events modulating these ethanol-responsive genes may also be involved in ethanol LORR behavior. 

Of the 32 genes identified by analysis of Fyn/LORR correlates of ethanol-responsive gene expression across *Fyn* null and recombinant inbred lines (Figure 4C), only 2 genes (*Cacna2d1* and *Ptprb*) were in common with the basal network (Figure 4B) other than Fyn kinase itself, suggesting the ethanol-responsive network is largely distinct from basal differences in expression. However, although the individual genes differed, this Fyn centric ethanol-responsive network was over-represented for functional categories related to ion channels (Table S7), as seen with basal Fyn network; notably including *Kcnj9*, *Cacnb4*, *Kcnma1*, and *Gabra2*. Several of these genes have been characterized for their association with ethanol-related phenotypes in mice and humans, although their association with Fyn kinase has not previously been identified. In particular, *Kcnj9* has been identified as a potential quantitative trait gene (QTG) for sedative-hypnotic withdrawal from ethanol [60], residing on mouse chromosome 1 syntenic to region of human chromosome 1 that has been identified for alcohol dependence [61-63]. Single nucleotide polymorphisms in *Gabra2* are associated with alcohol-elicited cues in the medial frontal cortical area [64], alcohol dependence [65], and acute effects of ethanol in humans [66]. Genetic knock-in of an ethanol insensitive mutant for *Gabra2* in mice causes increased acute ethanol-induced hypnosis, loss of motor stimulation, and altered ethanol-drinking behavior [67]. In respect to *Kcnma1*, acute ethanol is also known to modulate the voltage and Ca^++^ sensitivity of the BK potassium channel [68] causing acute intoxication in the model organism *Caenorhabditis elegans* [69]. 

**Figure 6:**
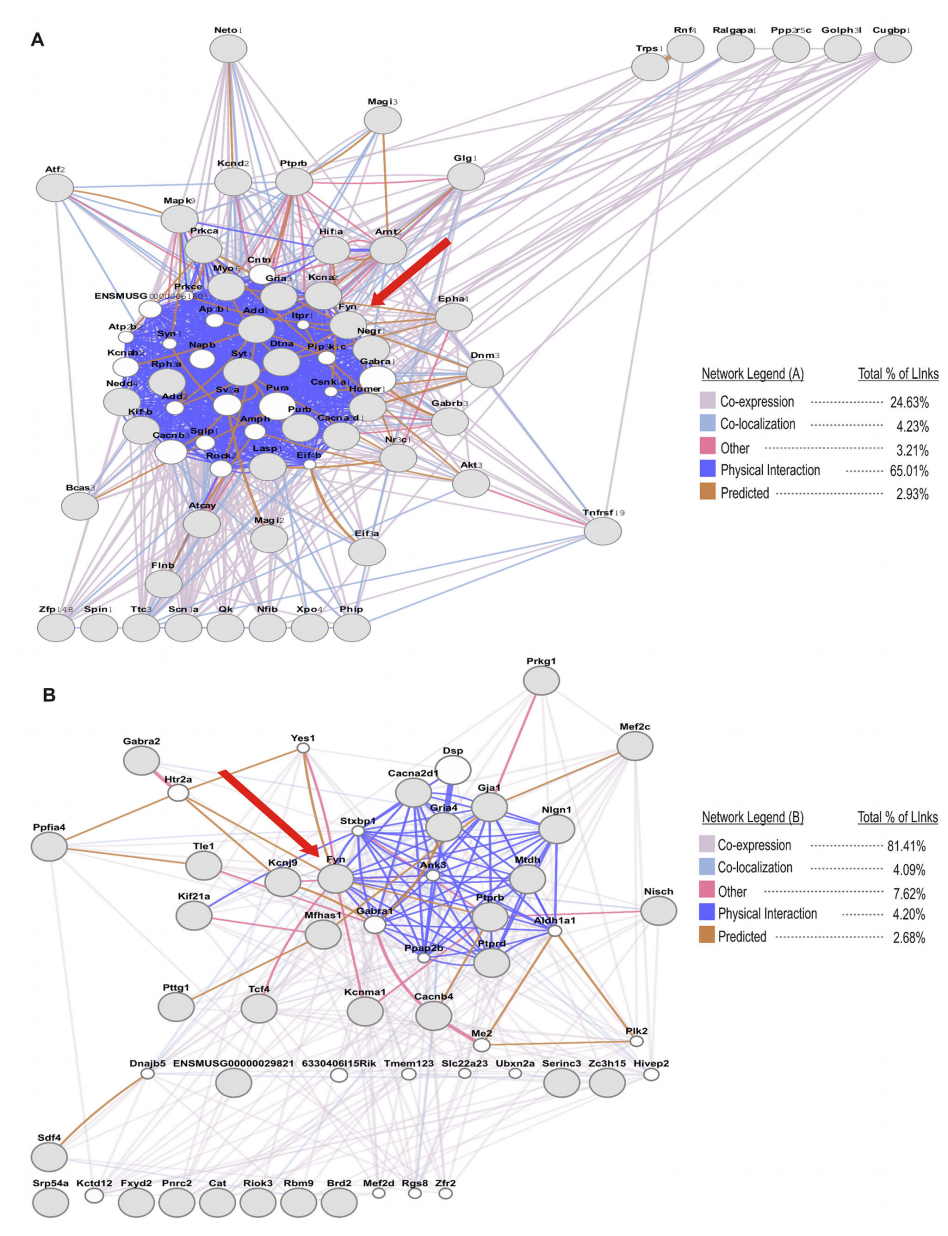
Independent assessment of Fyn kinase correlation networks. GeneMANIA web-tool (http://genemania.org/) analysis using public resources for Fyn-related gene expression networks: (A) Reconstruction of basal network from Figure 4B. (C) Reconstruction of ethanol-responsive network from Figure 4C. Grey nodes are query genes and white nodes are genes predicted form the GeneMANIA algorithm. Red arrow indicates the location of Fyn kinase.

The resulting Fyn-related basal and ethanol-responsive gene networks were submitted to GeneMANIA (www.genemania.org) [32] to both validate our networks across multiple external datasets related to gene co-expression, genetic interaction, and protein interaction, and perhaps identify network members missed in our analysis. The networks from GeneMANIA are qualitatively similar to our networks within PFC, suggesting these genes have tightly inter-related functional and regulatory mechanisms. Approximately 65% of all links between two genes within the basal network were due to protein-protein interactions (i.e. physical interactions) (Figure 6A). Although not a direct proof of causality, this GeneMANIA analysis thus provided strong *in silico* evidence supporting our microarray-derived Fyn-centric networks correlating with LORR behavior. The ethanol-responsive network (Figure 6B) was slightly different with approximately 81% of all connections derived from co-expression of genes; however, a subset of genes may physically interact with Fyn kinase. GeneMANIA datasets do not necessarily address gene-gene interactions following ethanol exposure and thus may underestimate the degree of physical interactions under our particular experimental conditions. 

GeneMANIA predicted the membership of multiple other candidate genes to both the basal and ethanol-responsive networks (Figure 6, nodes labeled in white). Notably, the analysis predicted the neurotrophic receptor *Ntrk2* and the phosphatase *Pten* within the basal network, and a subunit for the serotonin (*Htr2a*) and GABA-A (*Gabra1*) receptor within the ethanol-response network. All of these genes have been previously reported to have associations with the molecular and behavioral responses to ethanol. Thus, our analysis has defined a set of Fyn-related correlation networks from the measurement of steady state mRNA following either an acute dose of saline (basal) or ethanol. Overlapping these results with behavioral correlates of the loss of righting reflex across the BXD RI lines has led to the identification of Fyn-related gene networks that may have a functional role in acute sensitivity to ethanol, as seen with LORR. 

## Discussion

Fyn kinase has been reported previously to modulate the sedative-hypnotic effects of ethanol as determined by assessing LORR behavior [3-5]. This modulation of ethanol LORR has generally been ascribed to a direct role of Fyn kinase on modulation of ethanol-induced acute functional tolerance by phosphorylation of the NR2B subunit of NMDA receptors. Herein, we have conducted a genomic analysis of the mesocorticolimbic system from *Fyn* knockout mice in the presence and absence of ethanol to determine gene expression patterns associated with LORR that may have underlying associations beyond a single null mutation. Our results suggest several gene network level alterations in Fyn null mice that may also contribute to the modulation of ethanol-induced LORR, a behavioral phenotype characterized by both initial sensitivity and acute functional tolerance [70,71].

Our *in silico* analysis using the GeneNetwork resource showed that variation in the expression of *Fyn* within PFC is significantly correlated to a published report of the LORR across the BXD recombinant inbred strains of mice. Additionally, *Fyn* and LORR are correlated to a common set of genes altered in the *Fyn* knockout mouse, suggesting a *Fyn*-related network of genes influencing this behavioral phenotype. In agreement with previous research, our array analysis also suggested a deregulation of glutamatergic and GABAergic function in *Fyn* -/- mice; however, our analysis also suggest multiple other systems are perturbed in their expression, especially those related to myelin-associated gene expression. Our genomic study thus provides new insight as to the possible mechanisms of Fyn signaling in behavioral sensitivity to ethanol, and may contribute to the broader understanding of LORR and acute functional tolerance.

Basal abundance of myelin-associated gene expression was significantly disrupted in forebrain of *Fyn* knockout mice (Figure 2). Myelin gene and protein expression is impaired in the frontal cortex of alcoholics [72,73] and is suggested to be a dynamic aspect of substance abuse and comorbid disorders such as schizophrenia [74]. Differential expression of basal and acute ethanol-responsive myelin gene expression within medial PFC has also been previously suggested as an important aspect of lasting ethanol behavioral phenotypic differences between B6 and D2 mice [17]. Myelin gene expression has been shown to also be dynamically regulated by synaptic activity [75]. This suggests that these myelin gene network perturbations in *Fyn* null mice might actively alter neuronal signaling and plasticity. We thus hypothesize that variation of myelin-associated gene expression is a contributing factor in the sedative-hypnotic effects of ethanol and contributes to the previously documented changes in ethanol LORR with *Fyn* null mice. This also raises the possibility that variation in myelin gene expression might alter other behavioral responses to ethanol. Ongoing studies in our laboratory are aimed at exploring this possibility.

In addition to changes in myelin gene expression basally, the Fyn null mutation also produced considerable alterations in ethanol-responsive gene expression networks (see Figure 3 and Figure S1). Although limited to some degree by a single dose and time point of ethanol exposure, our targeted network analysis generates testable hypotheses for mechanistic studies on how the Fyn null mutation alters brain response to acute or chronic ethanol. Although beyond the scope of the current study, a full dose response and time course on ethanol-evoked changes in gene expression could have added insight on whether aspects of the genomic responses identified here might have a role in components of ethanol LORR, such as initial sensitivity and acute functional tolerance [70,71,76]. Similarly, studies on both male and female animals would have further broadened the implications of our studies. Whether these alterations in ethanol-responsive gene networks between the control vs. Fyn null animals are a direct downstream effect of Fyn signaling or due to more indirect developmental compensation issues within the knockout animals remains to be determined. Regardless, these alterations in ethanol-responsive gene expression could have important implications for downstream molecular and behavioral adaptive events occurring with chronic ethanol exposure.

Acute functional tolerance due to rapid molecular neuroadaptations to acute ethanol effects on brain signaling events is thought to play an important role in the duration of ethanol LORR [23,76]. Phosphorylation of the NMDA receptor subunit NR2B via Fyn kinase may account for one mechanism involved in this behavioral adaptation to acute ethanol [4,77]. Our analysis has focused exclusively on coordinate expression of steady-state mRNA and does not directly reflect phosphorylation events, other post-translation modifications, or changes in protein expression. Concordance between gene expression and protein levels is not always a simple linear relationship and may depend on the individual genes, local environment, preexisting conditions, the model organism in question, or protein half-life [78]. Although protein abundance and function are important for cellular machinery, transcript abundance may be more predictive of the overt phenotype and response to environmental stimuli [79]. 

In conclusion we have characterized a set of gene expression networks important in the modulation of acute ethanol sensitivity (LORR) within a *Fyn* knockout mouse. Our results are consistent with previous research related to Fyn kinase and ethanol behavioral phenotypes previously reported in the literature; however, they suggest a novel gene network perspective within individual brain regions contributing to altered acute ethanol sensitivity in *Fyn* kinase null mice. Convergent results from three different microarray studies and genes correlating with the LORR identified a significant network within PFC related to acute ethanol behavioral sensitivity. In particular, basal variation in gene expression identified a set of genes related through protein-protein interaction, including Fyn kinase. Thus these results suggest that Fyn interaction with a network of genes, including a group related to myelin structure and function, may modify the sedative-hypnotic properties of ethanol. Although not a prominent correlation in all published studies [80], the inverse relationship between acute ethanol sensitivity and the magnitude of long-term drinking behavior both in humans and animal models suggests that these Fyn-centric networks could also be part of the molecular factors influencing ethanol consumption, and possibly contribute to genetic mechanisms governing predisposition to AUD.

## Supporting Information

Figure S1 **Ingenuity pathway analysis networks for ethanol responsive gene expression in prefrontal Cortex (**A**), and ventral midbrain (**B**).** Upper panels show ethanol-responsive gene expression in controls and lower panels are from *Fyn* knockout animals. Genes labeled in green are down-regulated by acute ethanol; Red labeled genes are up-regulated by acute ethanol. Numbers shown are the mean S-score for 3 biological replicates. Qualitative differences in overall gene expression (C) are shown using the cumulative absolute S-scores for nucleus accumbens, prefrontal cortex, and ventral midbrain.
(TIFF)

Figure S2 **Venn Diagram of Fyn Networks.** (A) Venn diagram of basal datasets from Figure 4B; (B) Venn diagram of EtOH-response datasets from Figure 4C. Numbers shown represent unique gene symbols excluding *Fyn* itself. White = Fyn kinase array data, Blue = BXD *Fyn* correlates, Purple = LXS *Fyn* correlates, and Green = LORR gene expression correlates.
(TIFF)

Table S1 **Gene expression results for clustergram in Figure 1.** Values are the mean of 3 biological replicates within each brain region and treatment group.
(XLS)

Table S2 **Gene Ontology analysis of basal differences in gene expression between *Fyn* knockout mice and controls for NAC, PFC, and VMB.** Results are listed for Molecular Function, Biological Process, Cellular Component, and Mouse Phenotype using the ToppGene Suite at a 5% FDR and trimming groups for containing at least 3 and no greater than 300 members.
(XLS)

Table S3 **Gene Ontology analysis of ethanol-responsive differences in gene expression between *Fyn* knockout mice and controls for NAC, PFC, and VMB.** Results are listed for Molecular Function, Biological Process, Cellular Component, and Mouse Phenotype using the ToppGene Suite at a 5% FDR and trimming groups for containing at least 3 and no greater than 300 members.
(XLSX)

Table S4 **Gene list from analysis of Fyn or LORR expression correlations for basal expression data as described in Figure 4A.** Data are the 50 genes contained in Figure 4B network. Genes have significant correlations with basal Fyn expression in the PFC BXD and LXS datasets, correlate with ethanol LORR behavioral data and have altered basal expression in PFC between control and Fyn null animals. Values are Pearson correlations and corresponding p-values. Correlations were performed from databases within the GeneNetwork web resource.
(XLSX)

Table S5 **Functional over-representation analysis of basal gene expression correlations with Fyn expression or LORR from Figure 4B and Table S4.** Results are listed for Molecular Function, Biological Process, Cellular Component, and Mouse Phenotype using the ToppGene Suite at a 5% FDR and trimming groups for containing at least 3 and no greater than 300 members.
(XLSX)

Table S6 **Gene list from analysis of Fyn or LORR expression correlations for ethanol-responsive expression data as described in Figure 4A.** Data are the 32 genes contained in Figure 4C network. Genes have significant correlations with ethanol-responsive Fyn expression in the PFC BXD and LXS datasets, correlate with ethanol LORR behavioral data and have altered responsive expression in PFC between control and Fyn null animals. Values are Pearson correlations and corresponding p-values. Correlations were performed from databases within the GeneNetwork web resource.
(XLS)

Table S7 **Functional over-representation analysis of ethanol-responsive gene expression correlations with Fyn expression or LORR from Figure 4C and Table S5.** Results are listed for Molecular Function, Biological Process, Cellular Component, and Mouse Phenotype using the ToppGene Suite at a 5% FDR and trimming groups for containing at least 3 and no greater than 300 members.
(XLSX)
