# Supplementary figures and images for: Fyn-Dependent Gene Networks in Acute Ethanol Sensitivity
Source: PLoS One. 2013 Nov 29;8(11):e82435. doi: 10.1371/journal.pone.0082435 (PMC3843713; doi:10.1371/journal.pone.0082435)

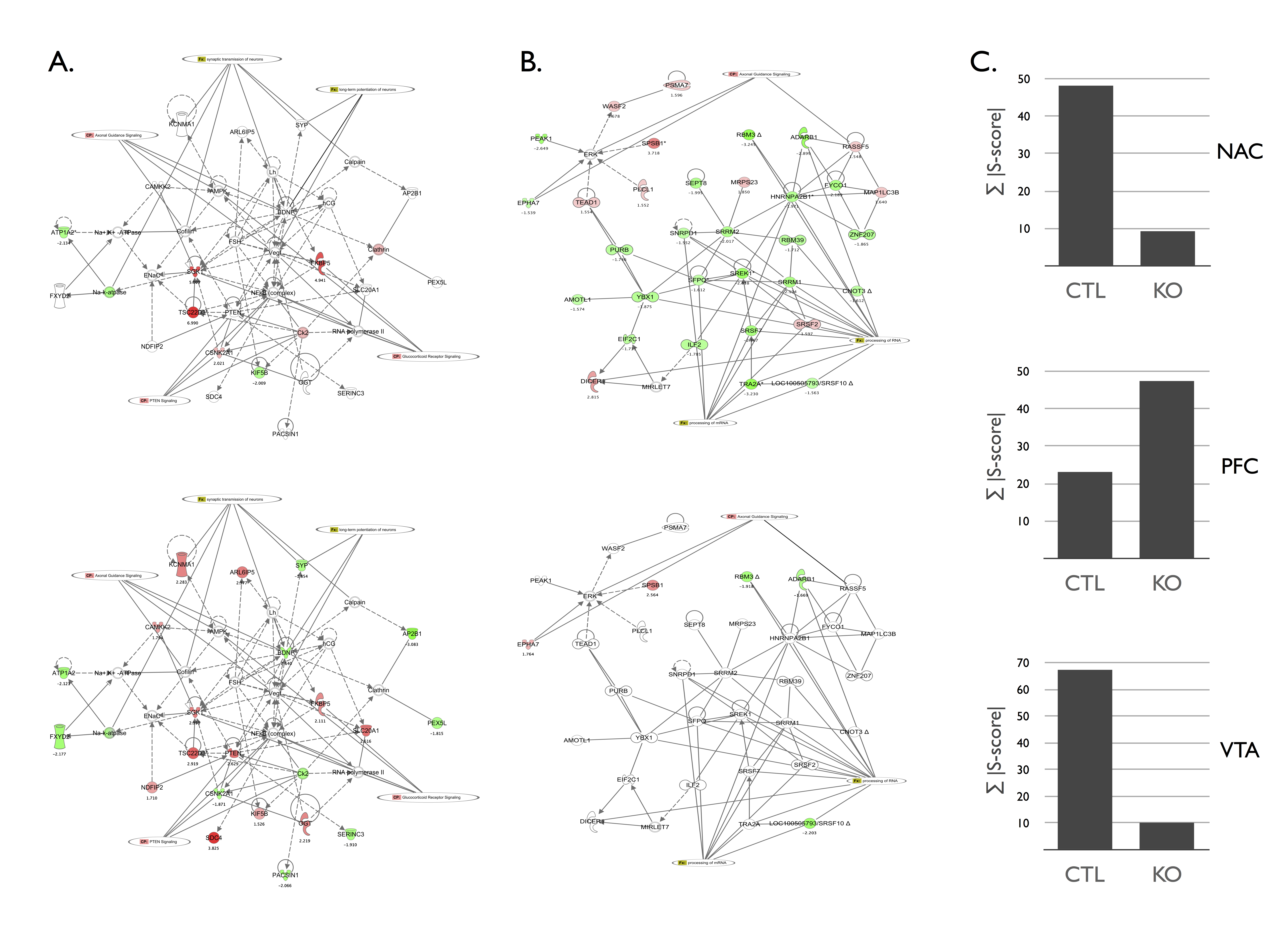

Supplement: Figure S1 — Ingenuity pathway analysis networks for ethanol responsive gene expression in prefrontal Cortex (A), and ventral midbrain (B). Upper panels show ethanol-responsive gene expression in controls and lower panels are from Fyn knockout animals. Genes labeled in green are down-regulated by acute ethanol; Red labeled genes are up-regulated by acute ethanol. Numbers shown are the mean S-score for 3 biological replicates. Qualitative differences in overall gene expression (C) are shown using the cumulative absolute S-scores for nucleus accumbens, prefrontal cortex, and ventral midbrain. (TIFF) [file pone.0082435.s001.tiff]

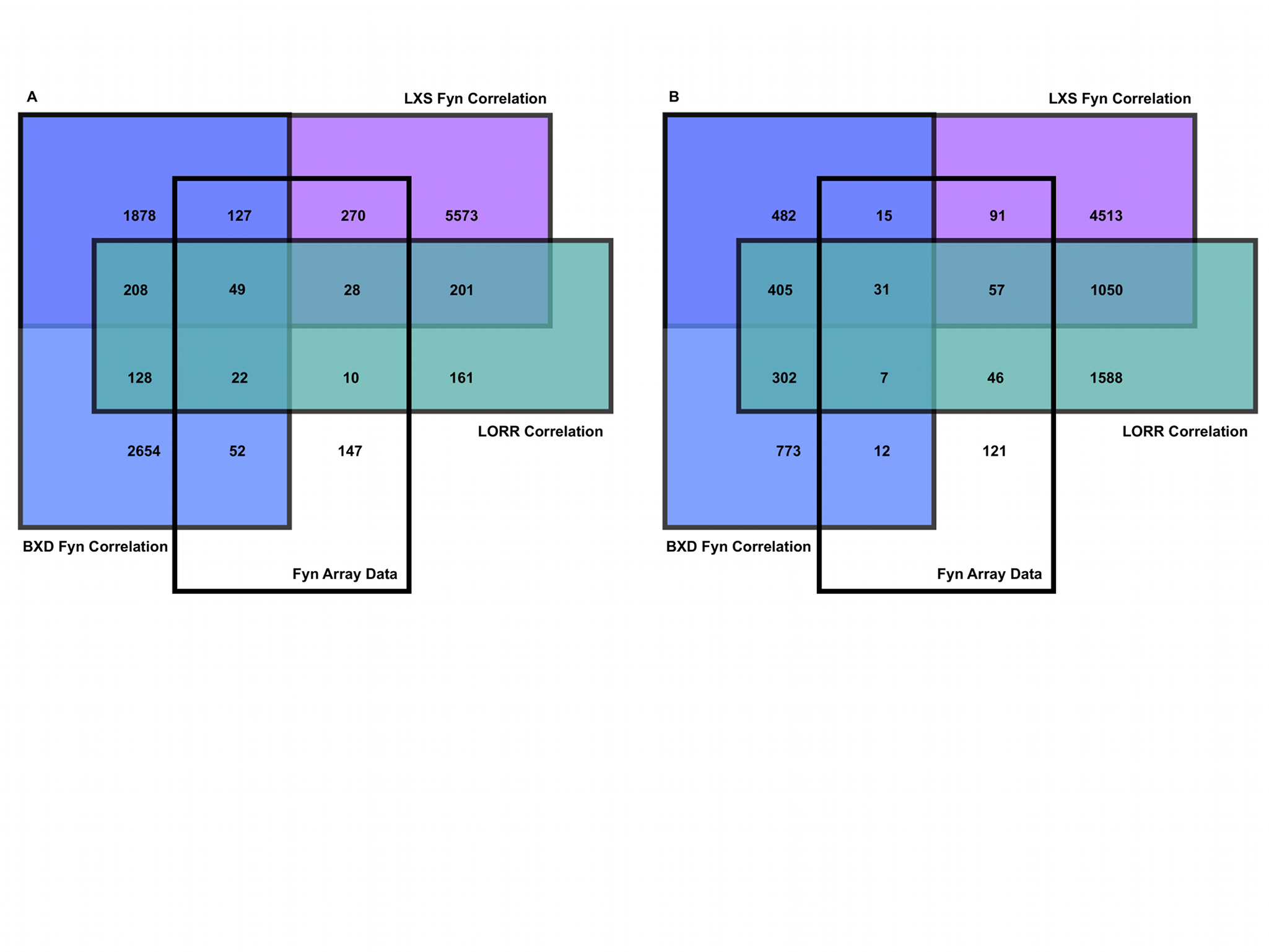

Supplement: Figure S2 — Venn Diagram of Fyn Networks. (A) Venn diagram of basal datasets from Figure 4B; (B) Venn diagram of EtOH-response datasets from Figure 4C. Numbers shown represent unique gene symbols excluding Fyn itself. White = Fyn kinase array data, Blue = BXD Fyn correlates, Purple = LXS Fyn correlates, and Green = LORR gene expression correlates. (TIFF) [file pone.0082435.s002.tiff]
